# Alectinib versus crizotinib in ALK‐positive advanced non‐small cell lung cancer and comparison of next‐generation TKIs after crizotinib failure: Real‐world evidence

**DOI:** 10.1002/cam4.4834

**Published:** 2022-05-26

**Authors:** Yurong Wang, Shujing Shen, Peizhu Hu, Di Geng, Ruipan Zheng, Xingya Li

**Affiliations:** ^1^ Department of Medical Oncology The First Affiliated Hospital of Zhengzhou University Zhengzhou Henan Province People's Republic of China; ^2^ Department of Radiotherapy The First Affiliated Hospital of Zhengzhou University Zhengzhou Henan Province People's Republic of China; ^3^ Department of Pathology The First Affiliated Hospital of Zhengzhou University Zhengzhou Henan Province People's Republic of China

**Keywords:** alectinib, ALK, brigatinib, ceritinib, crizotinib, PFS

## Abstract

**Background:**

Anaplastic lymphoma kinase (ALK) fusion is a prognostic indicator for patients with non‐small cell lung cancer (NSCLC) receiving tyrosine kinase inhibitors (TKIs). The real‐world data of ALK TKIs remain a major concern.

**Methods:**

Patients with ALK‐positive advanced NSCLC, who received crizotinib or alectinib treatment in first line, were retrospectively reviewed. ALK status was detected using immunohistochemistry (IHC) or next‐generation sequencing (NGS). Clinical outcomes have been comprehensively analyzed between TKIs, ALK fusions, EML4‐ALK variants, and next‐generation TKIs after crizotinib failure.

**Results:**

One hundred sixty‐eight patients were successively enrolled (crizotinib, *n* = 109; alctinib, *n* = 59). Alectinib showed consistent superiority in progressive‐free survival (PFS) over crizotinib (hazard ratio [HR]: 0.43, 95% confidential interval [CI]: 0.24–0.77, *p* = 0.004). Multivariate Cox regression showed chemotherapy (CT) prior to TKIs or synchronous chemotherapy seemed not to improve PFS compared to ALK inhibitors alone (*p* > 0.05). And, alectinib was superior to crizotinib in prolonging intracranial PFS (HR 0.12, 95% CI: 0.03–0.49, *p* = 0.003). Patients in EML4 group had a better prognosis than those in non‐EML4 group after alectinib administration (HR 0.13, 95% CI: 0.03–0.60, *p* = 0.009). TP53 co‐mutations were relatively common (34.0%) and associated with adverse outcome in ALK‐positive patients (adjusted HR 2.22, 95% CI: 1.00–4.92, *p* = 0.049). After crizotinib failure, 33 patients received a sequential application of next‐generation ALK TKIs. Compared to ceritinib and brigatinib, alectinib might have better PFS (*p* = 0.043).

**Conclusion:**

Our results revealed alectinib had better PFS and higher intracranial efficacy compared to crizotinib in ALK‐positive NSCLC, and might improve PFS by comparison with ceritinib and brigatinib after crizotinib failure.

## Graphical abstract

1. Chemotherapy prior to TKI or in combination with TKI showed no improvements in PFS compared to ALK inhibitors alone. 2. Alectinib showed better PFS and higher intracranial efficacy by comparison with crizotinib, especially in patients with fusion involving EML4‐ALK. 3. After failure of crizotinib, alectinib might have better PFS compared to ceritinib and brigatinib. 4. TP53 co‐mutations were relatively common and associated with adverse outcome of ALK‐positive NSCLC.

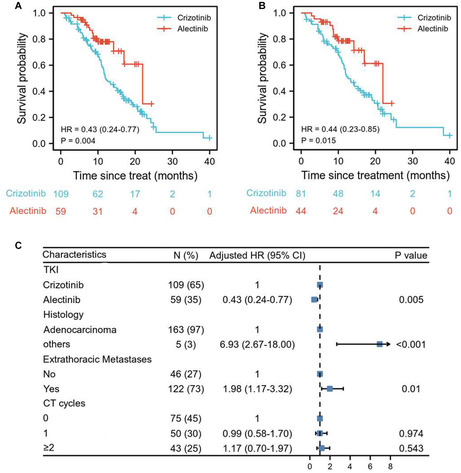

## 1 INTRODUCTION

Anaplastic lymphoma kinase (ALK)‐rearrangement (also known as ALK fusion) accounts for 3%–11% in non‐small cell lung cancer (NSCLC) which is the leading cause of cancer‐related death in China and worldwide.^1^, ^2^ ALK can rearrange with a variety of genes, which activates signal transduction pathways related to cell proliferation, leading to carcinogenesis. Echinoderm microtubule‐associated protein‐like 4 (EML4) is the most common partner of ALK fusion, as well as PTPN3, KIF5B, TFG, KLC1, STRN, TPR, and HIP1, and so on. Among the EML4‐ALK variants, variant 1 (E13, A20) and variant 3a/b (E6, A20) are the most frequent EML4‐ALK types.^3^ And previous studies demonstrated that differential ALK fusions and EML4‐ALK variants had differential responses to tyrosine kinase inhibitor (TKI).^3^

Solomon and colleagues have proved that crizotinib was superior to the platinum‐based chemotherapy.^4^ Then the Food and Drug Administration (FDA) approved crizotinib as the first TKI for ALK‐positive patients with advanced NSCLC in 2011, followed by its approval of the National Medical Products Administration (NMPA) in 2013. Due to the poor penetration of blood–brian barrier (BBB), crizotinib treatment is less effective against intracranial metastases. Besides ALK fusion, crizotinib can also target ROS1 fusion, MET amplification or MET exon 14 skipping, resulting in more toxicity‐related complications and discontinuation of current treatment. To address these issues, more effective next‐generation TKIs have been developed, such as alectinib, ceritinib, brigatinib, lorlatinib, and so on. Results from ALEX study showed alectinib brought ALK‐positive patients a longer progression‐free survival (PFS) than crizotinib (median PFS, 34.8 months vs. 10.9 months).^5^ Ceritinib, a second‐generation ALK TKI, has been shown to inhibit ALK about 20 times as much as crizotinib in vitro studies. The clinical trials of ASCEND series demonstrated that ceritinib was significantly superior to chemotherapy (median PFS, 5.4 months vs. 1.6 months) in patients with advanced ALK‐positive NSCLC resistant to crizotinib treatment and had a good safety profile.^6^, ^7^ Therefore, alectinib and ceritnib have been approved by NMPA for managing NSCLC patients resistant or intolerant to crizotinib in 2018. Moreover, alectinib was approved as first line treatment for ALK‐positive patients.

However, patients in prospective clinical trials were selected. What about their data in real‐world? Given that, we performed this retrospective study and analyzed the clinical outcomes between crizotinib and alectinib, and the survival benefits among the subsequent ALK TKIs when failed to crizotinib treatment. In addition, the efficacy of TKIs among different ALK fusions, EML4‐ALK variants, as well as the impact of TP53 co‐mutation on survival of ALK‐positive patients was also explored.

## 2 MATERIALS AND METHODS

### 2.1 Patients

One hundred and sixty‐eight ALK‐positive local advanced or metastatic NSCLC patients were retrospectively reviewed and enrolled successively from our hospital between March 2017 and September 2020. The inclusion criteria were as follows: (1) ALK rearrangement confirmed by Ventana ALK (D5F3) CDx IHC assay or NGS; (2) stage IIIB, IIIC, and IV disease according to the 8th American Joint Committee on Cancer Staging System; (3) administration of crizotinib 250 mg (from March 2017 to August 2020) or alectinib 600 mg (from November 2018 to September 2020), twice daily, including those received prior or synchronous chemotherapy; (4) radiographic data available for evaluation. The exclusion criteria were as follows: (1) patients with other actionable driver alterations; (2) patients receiving systemic treatment other than TKIs and chemotherapy.

The baseline characteristics were reviewed and collected from the electronic records, including age, sex, histology, diagnosis, ALK status by IHC or NGS, treatment process, radiographic document, and so on. The best changes in tumor from baseline, response rates, and progression‐free survival (PFS) were analyzed according to Response Evaluation Criteria in Solid Tumors (version 1.1). The Ethics Committee of the First Affiliated Hospital of Zhengzhou University approved this retrospective analysis and waived informed consent.

### 2.2 IHC and NGS

The expression of ALK protein of FFRE sections was evaluated by Ventana ALK (D5F3) CDx IHC assay (Roche Diagnostics) according to the manufacturer's protocols. Tumor cells presented with strong granular cytoplasmic staining are defined as ALK‐positive case, while tumor cells without strong cytoplasmic staining are defined as ALK‐negative case.

The NGS details were described in our previous reports.^8^ Briefly, DNA was extracted from tumor tissues for NGS library construction. For sequencing, hybrid capture‐based probes targeting 8, 14, 56, or 425 gene (GeneseeqOne, Nanjing Geneseeq Technology Inc.; Burning Rock Biotech Ltd.) were adopted on Illumina HiSeq 4000 (Illumina). The sequencing depth was 1000 × in tissue sample at least. The below driver genes were generally covered in all the above panels: EGFR, ALK, KRAS, BRAF, ROS1, RET, MET, and ERBB2. Of note, only 56‐ and 425‐gene panel covered TP53 status. FACTERA software was used for genomic fusions.^9^

### 2.3 Statistical analysis

The baseline features and response rates were compared by chi‐square test or fisher‐exact test when chi‐square test was unsuitable. The survival analysis was performed using Kaplan–Meier method and Log‐rank test. Multivariate Cox regression model was performed for the analysis of prognostic factors. Two‐sided *p* values <0.05 were considered statistically significant. Data were also analyzed by SPSS (version 19).

## 3 RESULTS

### 3.1 Baseline characteristics of patients with ALK‐positive NSCLC

Among 168 patients diagnosed as advanced NSCLC, 109 patients received crizotinib and 59 patients received alectinib treatment. 54.1% (59/109) cases were treatment‐naive in crizotinib group, more than that in alectinib group with 27.1% (16/59), *p* < 0.001. Patients with 1 cycle of chemotherapy before ALK TKI accounted for 20.2% (22/109) in crizotinib group and 47.5% (28/59) in alectinib group. The proportion of CT cycles more than 2 was similar between the two groups (25.7% vs. 25.4%). The other clinical baseline characteristics such as age, sex, ECOG PS, smoke history, histology, TNM stage, extrathoracic metastases (brain, liver, and bone metastases), ALK detection method, brain radiotherapy at preliminary diagnosis were not significantly different between crizotinib and alectinib group (Table 1).

**TABLE 1:** Baseline characteristics of patients with ALK‐positive NSCLC treated with crizotinib or alectinib

Characteristics	Crizotinib (*N* = 109)	Alectinib (*N* = 59)	*p*‐value
Age, years	53.1 ± 11.9	52.0 ± 12.2	0.633
Sex			0.997
Male	61 (56.0%)	33 (55.9%)	
Female	48 (44.0%)	26 (44.1%)	
ECOG PS			0.073
0–1	92 (84.4%)	43 (72.9%)	
2–3	17 (15.6%)	16 (27.1%)	
Smoke history			0.435
No	85 (78.0%)	49 (83.1%)	
Yes	24 (22.0%)	10 (16.9%)	
Histology			0.795
Adenocarcinoma	105 (96.3%)	58 (98.3%)	
Squamouscarcinoma	2 (1.8%)	1 (1.7%)	
Others	2 (1.8%)	0 (0.0%)	
TNM stage			0.733
III	11 (10.1%)	5 (8.5%)	
IV	98 (89.9%)	54 (91.5%)	
Extrathoracic metastases			0.435
No	32 (29.4%)	14 (23.7%)	
Yes	77 (70.6%)	45 (76.3%)	
Brain metastases			0.941
No	77 (70.6%)	42 (71.2%)	
Yes	32 (29.4%)	17 (28.8%)	
Liver metastases			0.081
No	84 (77.1%)	52 (88.1%)	
Yes	25 (22.9%)	7 (11.9%)	
Bone metastases			0.225
No	66 (60.6%)	30 (50.8%)	
Yes	43 (39.4%)	29 (49.2%)	
Detection method			0.443
IHC	29 (26.6%)	19 (32.2%)	
NGS	80 (73.4%)	40 (67.8%)	
8‐gene	28 (25.7%)	7 (11.9%)	
14‐gene	22 (20.2%)	16 (27.1%)	
56‐gene	7 (6.4%)	6 (10.2%)	
425‐gene	23 (21.1%)	11 (18.6%)	
VAF, %	11.6 ± 13.7	9.1 ± 10.1	
Fusion type			0.295
EML4‐ALK	61 (56.0%)	35 (59.3%)	
non‐EML4‐ALK	10 (9.2%)	3 (5.1%)	
Complex fusion involving EML4‐ALK	8 (7.3%)	1 (1.7%)	
Unknown	30 (27.5%)	20 (33.9%)	
Brain radiotherapy			0.335
No	104 (95.4%)	58 (98.3%)	
Yes	5 (4.6%)	1 (1.7%)	
CT cycles before or during TKI treatment			<0.001
0	59 (54.1%)	16 (27.1%)	
1 (before TKI)	22 (20.2%)	28 (47.5%)	
2 to 6	25 (22.9%)	12 (20.3%)	
≥7 (during TKI)	3 (2.8%)	3 (5.1%)	

Abbreviations: CT, Chemotherapy; ECOG PS, Eastern Cooperative Oncology Group performance status; VAF, variant allele fraction.

### 3.2 Effects between alectinib and crizotinib

The objective response rate (ORR) and disease control rate (DCR) to crizotinib and alectinib were estimated (Table S1–S3). And no significant difference was observed between the two cohorts (ORR: 67.0% vs. 74.6%, *p* = 0.306; DCR 95.4% vs. 98.3%, *p* = 0.666).

**FIGURE 1:**
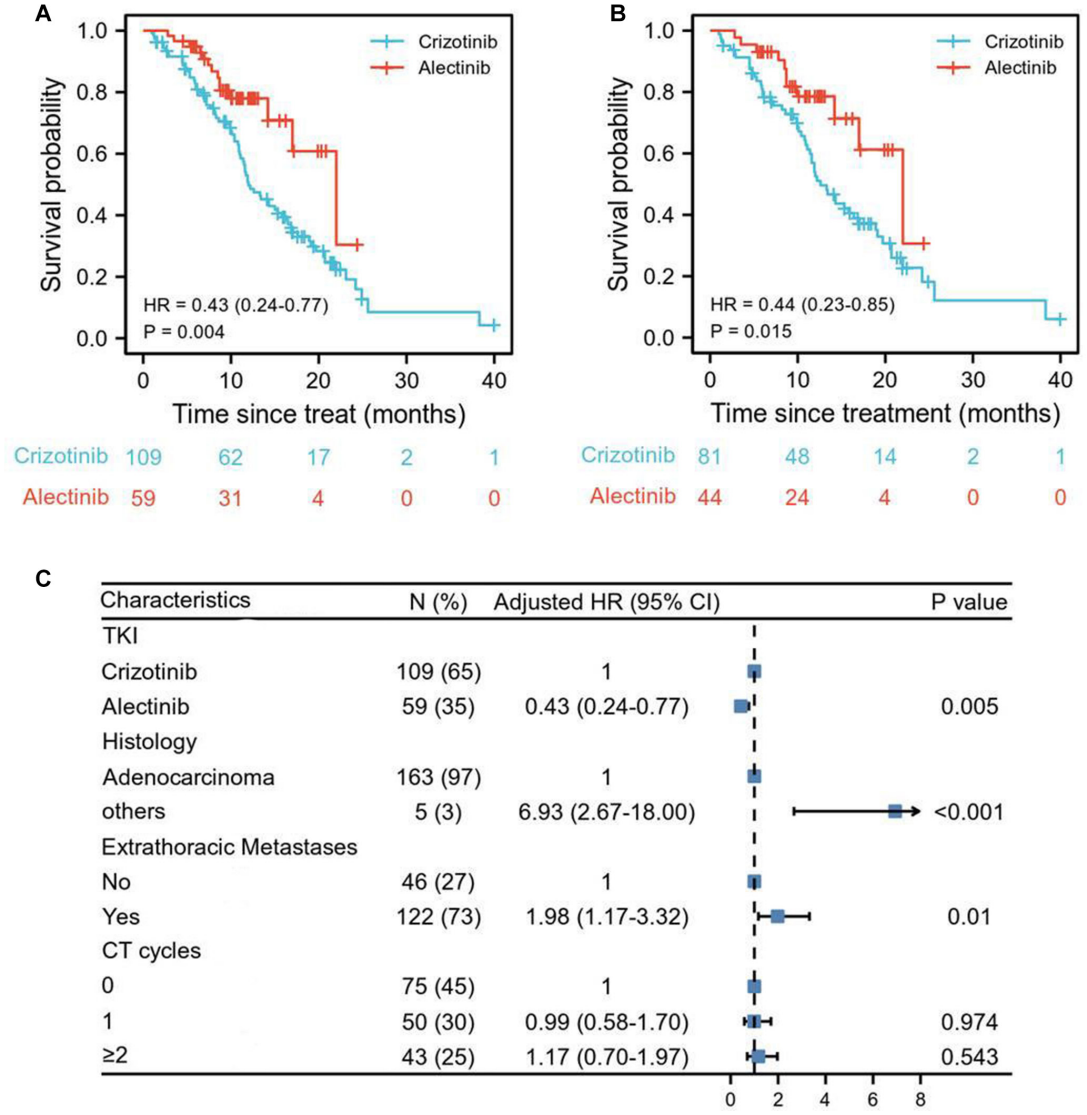
Alectinib showed consistent superiority in PFS over crizotinib. Kaplan–Meier curves were drawn for comparing the PFS between alectinib and crizotinib in all patients (A) and patients receiving no or only 1 cycle of chemotherapy prior to TKI (B). Prognostic factors for PFS were further analyzed using multivariate Cox regression model in all patients (C).

At the time of analysis, 23.7% (14/59) and 67.9% (74/109) events of disease progression were observed in alectinib and crizotinib group for the whole TKI‐naive population (Table S1–S3). Alectinib showed consistent superiority in PFS over crizotinib (HR: 0.43, 95% CI: 0.24–0.77, *p* = 0.004, Figure 1A). Similar results were obtained among the population (*n* = 125) receiving no or only 1 cycle of chemotherapy prior to TKI use (HR: 0.44, 95% CI: 0.23–0.85, *p* = 0.015, Figure 1B). The PFS rates of 6, 12, 18, and 24 months in alectinib group were all significantly better than that in crizotinib group (*p* < 0.05; Table S1–S3). Chemotherapy prior to TKIs or synchronous chemotherapy showed no improvements in PFS compared to ALK inhibitors alone (CT 1 cycle, HR 0.99, 95% CI: 0.58–1.70, *p* = 0.974; CT ≥ 2 cycles, HR 1.17, 95% CI: 0.70–1.97, *p* = 543, Figure 1C). Survival curves of patients receiving chemotherapy or not in crizotinib and alectinib groups were also described, respectively (Figure S1). Multivariate Cox regression analysis indicated that alectinib treatment (HR: 0.43, 95% CI: 0.24–0.77, *p* = 0.005), extrathoracic metastases (HR: 1.98, 95% CI: 1.17–3.32, *p* = 0.010), non‐adenocarcinoma (HR: 6.93, 95% CI: 2.67–18.00, *p* < 0.001) were the independent prognostic factors for PFS (Figure 1C).

### 3.3 Intracranial and extracranial outcomes

**FIGURE 2:**
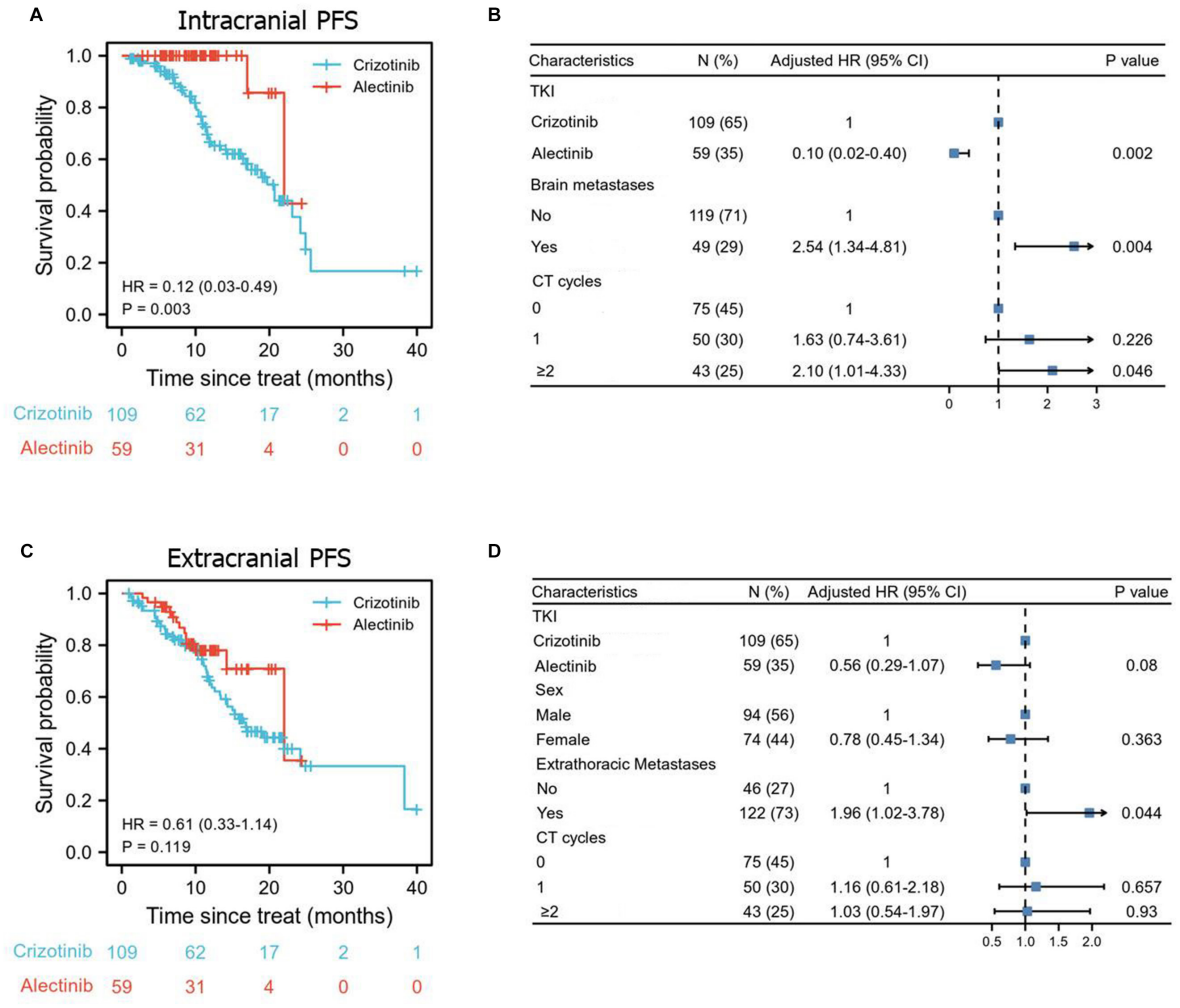
Alectinib showed higher intracranial efficacy by comparison with crizotinib. Intracranial PFS (A) and extracranial PFS (C) were described by Kaplan–Meier curves between crizotinib and alectinib in patients with ALK‐positive NSCLC. Prognostic factors for intracranial PFS (B) and extracranial PFS (D) were further analyzed using multivariate Cox regression model.

It is universally acknowledged that alectinib was superior to crizotinib in BBB penetration, but is it also more effective on extracranial metastases? Consistent with the previous studies, alectinib was still better than crizotinib in prolonging intracranial PFS (HR 0.12, 95% CI: 0.03–0.49, *p* = 0.003; Figure 2A). Alectinib treatment (HR: 0.10, 95% CI: 0.02–0.40, *p* = 0.002), brain metastases (HR: 2.54, 95% CI: 1.34–4.81, *p* = 0.004), and CT more than 2 cycles (HR: 2.10, 95% CI: 1.01–4.33, *p* = 0.046; Figure 2B) were independent predictors identified by multivariate cox regression analysis for intracranial PFS (Figure 2B). However, crizotinib exhibited a similar extracranial efficacy to alectinib as shown in Figure 2C,D (HR: 0.61, 95% CI: 0.33–1.14, *p* = 0.119).

### 3.4 Outcomes of different ALK fusions and EML4‐ALK variants

**FIGURE 3:**
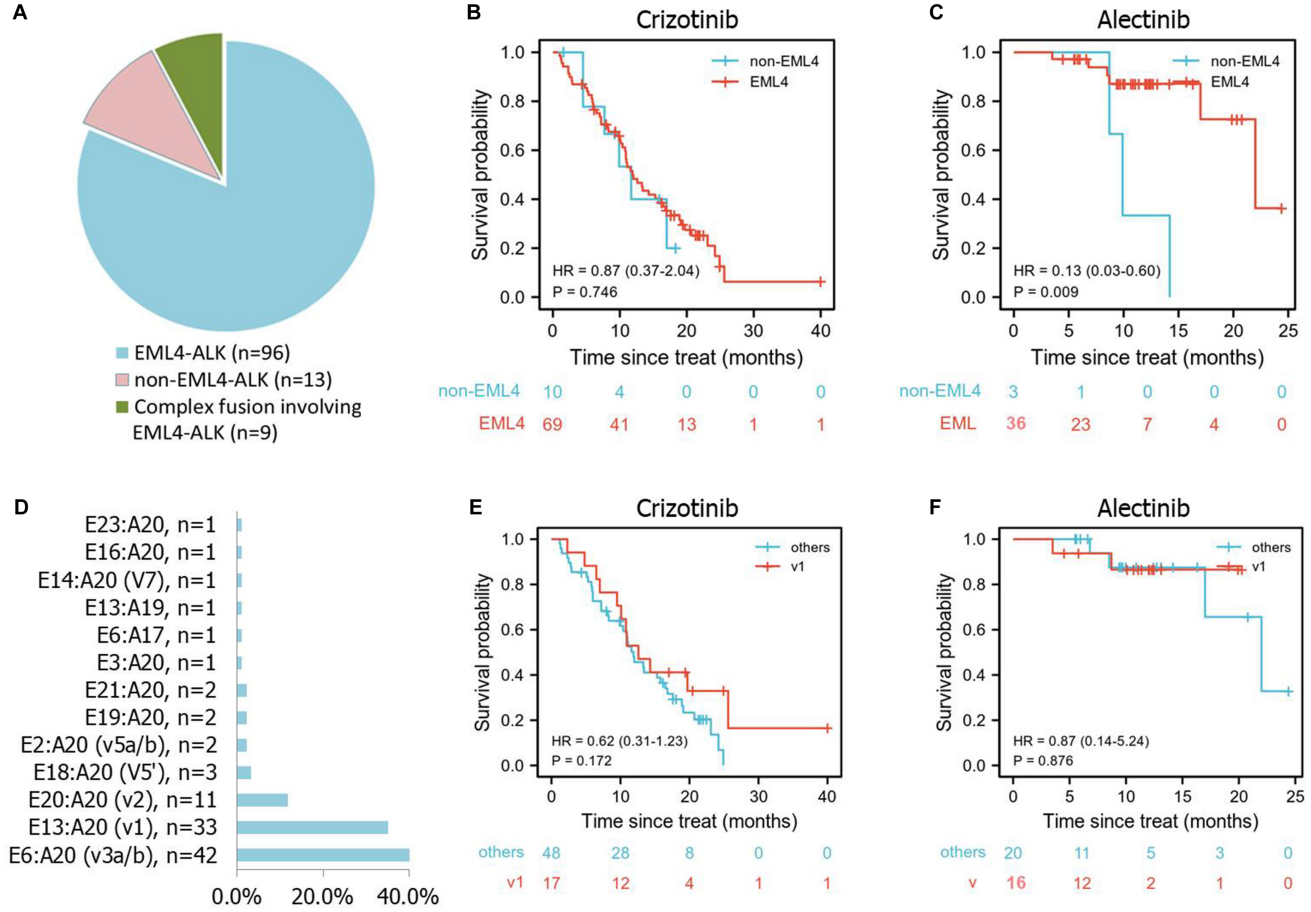
The effects of ALK TKIs on different ALK fusions and variants. The distribution of ALK fusion types (A) and EML4‐ALK variants (D); PFS comparison between subgroups of patients with different ALK fusions: EML4 group and non‐EML4 group in crizotinib cohort (B) and alectinib cohort (C); (E and F) PFS comparison between subgroups of patients with different EML4‐ALK variants: v1 versus others in crizotinib cohort (E) and alectinib cohort (F).

In total of 25 fusion partners were detected in our study and listed in Table 2, including EML4, ZNF362, TPM3, and so on. Among them, pure EML4‐ALK fusion is the most common subtype accounting for 81.3% (96/118), followed by other fusions except EML4‐ALK (non‐EML4 group) 11.0% (13/118) and complex fusions involving EML4‐ALK 7.6% (9/118) (Figure 3A). Crizotinib showed similar PFS benefits between EML4 group (including EML4‐ALK and complex fusion) and non‐EML4 group (HR 0.87, 95% CI: 0.37–2.04, *p* = 0.746, Figure 3B). However, alectinib prolonged the PFS of EML4 group compared to non‐EML4 group (HR 0.13, 95% CI: 0.03–0.60, *p* = 0.009, Figure 3C). For patients with uncommon fusion partners, the responses to ALK inhibitors and survival data are also listed in Table 2.

**TABLE 2:** The data of response to ALK inhibitors in patients with uncommon fusion partners

No.	Sex	Age	Partner	VAF, %	1st Line	Best Response	TST 1 (months)	2nd line	Best response	TST 2 (months)
22	M	56	ZNF362	4.4	Alectinib	PR^a^	9.9	Clinical trial	_	_
32	M	52	TPM3	12.2	Crizotinib	SD	18.4	_	_	_
34	F	51	CLTC	44.4	Crizotinib	PR^a^	11.7	Ceritinib	SD^a^	3.5
53	F	55	TRAF3/AE000662.92	6.94/6.44	Alectinib	PR^a^	8.7	chemotherapy	SD^a^	2.4
54	M	47	LINC01247	3.72	Crizotinib	PD	4.5	UN	_	_
59	M	67	MYH10	13.2	Crizotinib	SD^a^	9.9	Ceritinib	PR	23.0
65	F	37	ST6GAL2RGPD4	4.1	Crizotinib	PR	9.2	_	_	_
72	M	38	NEGR1/LINC01360	10	Crizotinib	PR^a^	4.5	Ceritinib	SD	4.2
75	M	59	NEB	5.1	Crizotinib	PR^a^	7.7	Alectinib	PR	26.8
102	M	31	MAP4K3	5.6	Crizotinib	PD	1.6	Brigatinib	SD	4.4
106	F	72	IGR(C16orf47)	6.4	Crizotinib	SD	15.9	_	_	_
123	M	45	PLXNA‐4	7.9	Crizotinib	PR^a^	17	Clinical trial	_	_
146	F	20	HIP1	39.1	Alectinib	PR^a^	14.2	UN	_	_
36	F	65	EML4/THADA	7.8/11.6	Alectinib	SD	20.8	_	_	_
62	F	45	EML4/CLIP4	18.29/22.95	Crizotinib	PR^a^	5.8	Ceritinib	PD	1.0
79	M	74	EML4/TLK2	17.79/24.84	Crizotinib	PR^a^	6	UN	_	_
81	M	62	EML4/LINC01317	19.9/17.4	Crizotinib	SD^a^	10	Brigatinib	SD^a^	5.0
86	M	37	EML4/NRXN1	26.6/20.6	Crizotinib	PD	1.2	chemotherapy	PR	4.2
97	M	63	EML4/DPF1&PPP1R14A	8.3/9.5	Crizotinib	PR	24.9	_	_	_
107	M	50	EML4/ABCA3	6.04/3.25	Crizotinib	SD^a^	5.3	UN	_	_
110	M	62	EML4/TCF7L1	5.42/7.03	Crizotinib	PR	21.8	_	_	_
125	F	51	EML4/PPP1R21	28.4/26.7	Crizotinib	SD	9.3	_	_	_

*Note*: TNM stages of all patients were IV, except that of patient 53 was III. The histology of all patients was adenocarcinoma, except that of patient 86 was squamous carcinoma.

Abbreviations: ICI indicates immune check point; PD, progressive disease; PR, Partial response; SD, Stable disase; TST, Time since treatment; UN, Unknown; VAF, variant allele fraction.

^a^ Patients had progressive diseases after responding to ALK inhibitors.

Among EML4‐ALK fusion, 13 variants in total were identified in this analysis (Figure 3D). The variant v3a/b (E6, A20) is the most frequent subtype with a proportion of 41.6% (42/101), followed by v1 (E13, A20) with 32.7% (33/101) and v2 (E20:A20) with 10.9% (11/101). No significant difference was observed between v1 and non‐v1 groups treated by either crizotinib or alectinib (Figure 3E,F).

### 3.5 Impact of TP53 co‐mutation on PFS

**FIGURE 4:**
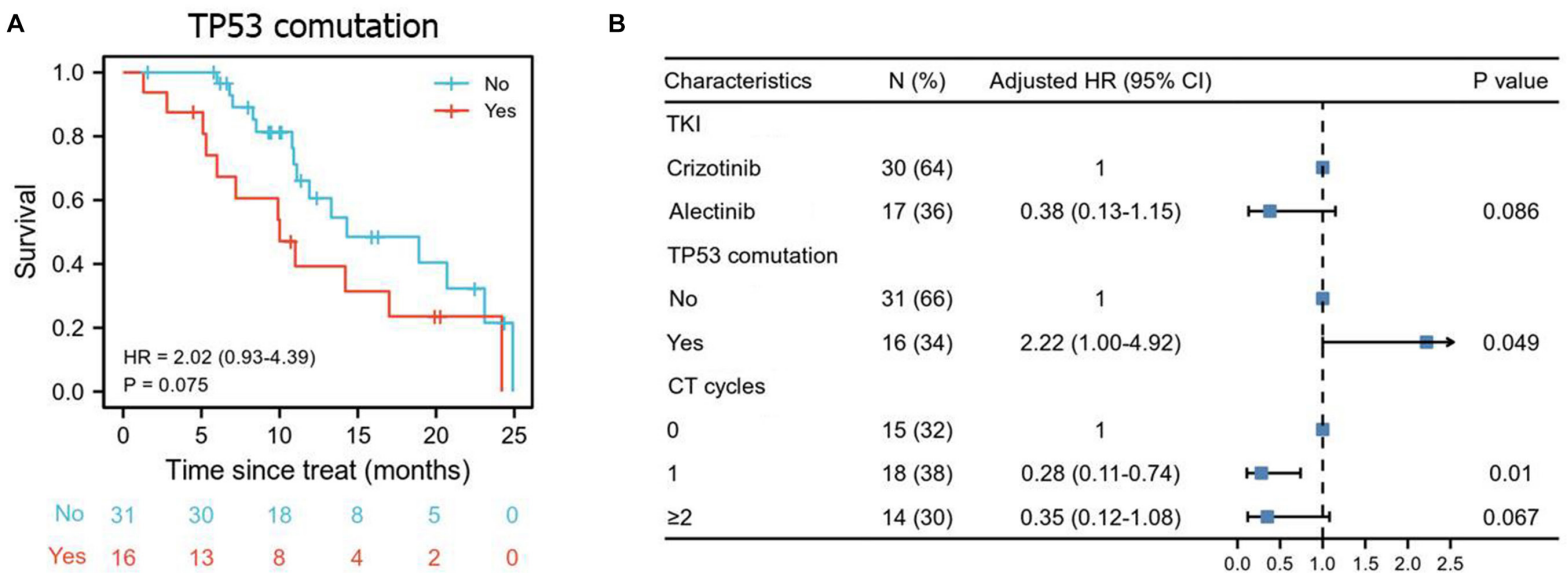
The influence of TP53 co‐mutation on PFS of ALK‐positive NSCLC. PFS analysis of TP53 co‐mutation (A), prognostic factors for PFS under multivariate Cox regression analysis (B).

How about the impact of TP53 co‐mutation on survival of ALK‐positive patients? We performed the analysis on 47 patients who were profiled by 56‐ and 425‐gene NGS covering TP53 gene. The results showed that patients with TP53 co‐mutations were relatively common accounting for 34.0% (16/47) and tended to have a poorer survival than those without (HR: 2.02, 95% CI: 0.93–4.39, *p* = 0.075), which was confirmed by multivariate cox‐regression analysis (adjusted HR 2.22, 95% CI: 1.00–4.92, *p* = 0.049) (Figure 4).

### 3.6 Effects of next‐generation TKIs at progression on crizotinib

Prospective clinical studies have shown that both ceritinib and alectinib have a good effect on crizotinib‐resistant ALK‐positive patients, but what about their effects in real‐world? In our study, 33 patients received next‐generation TKIs after failure of crizotinib. The baseline characteristics were not significantly different, such as age, sex, ECOG PS, smoke history, histology, TNM stage, ALK detection method, fusion type after disease progression (Table S2).

**FIGURE 5:**
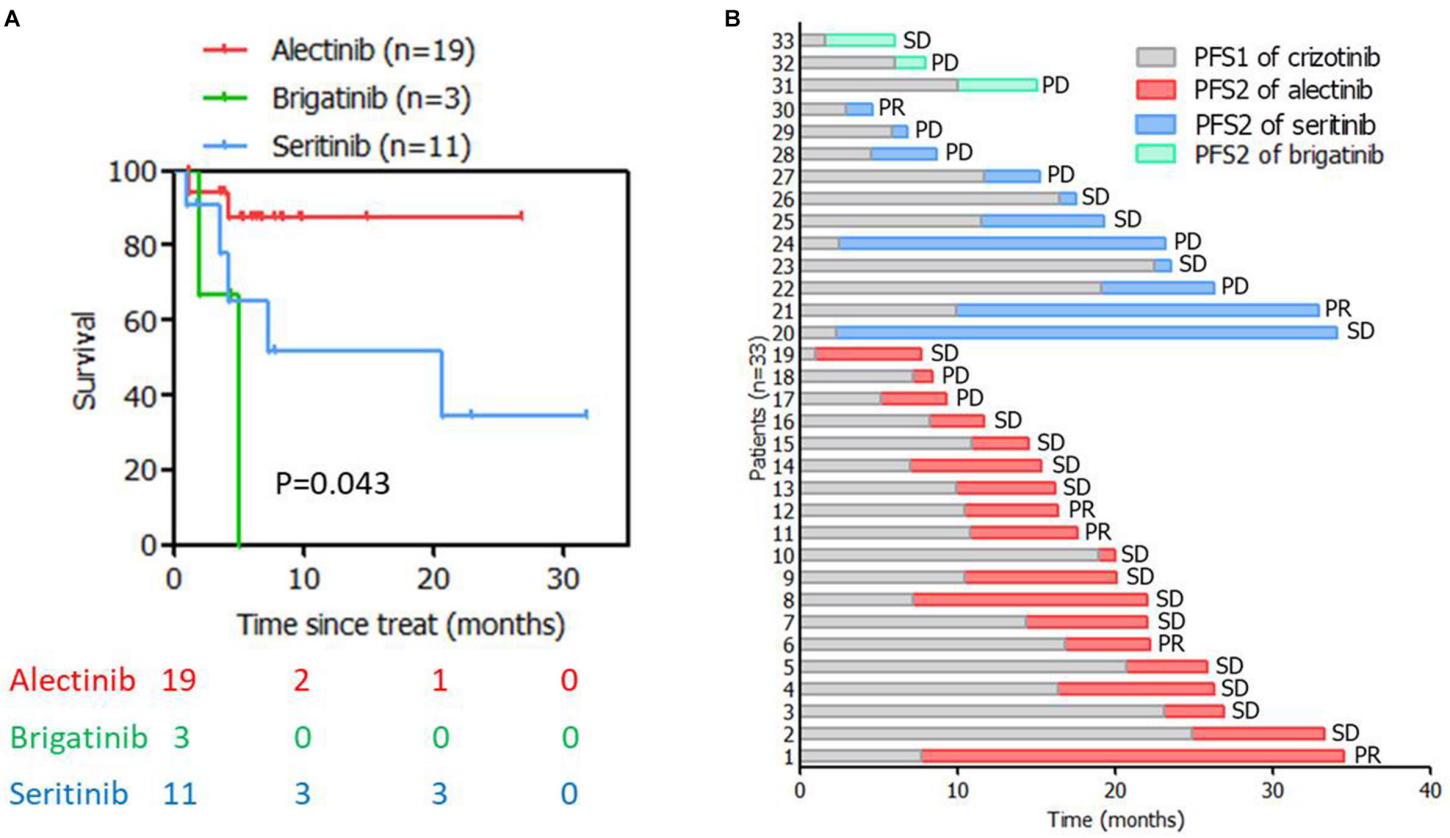
PFS of next‐generation of ALK TKIs after crizotinib failure. PFS comparison (A) and stacked column graph of PFS2 of next‐generation of ALK TKIs (B).

The ORR and DCR were similar between alectinib, ceritinib, and brigatinib (ORR: 26.3% vs. 27.3% vs. 0.0%, *p* = 0.589; DCR 94.7% vs. 90.0% vs. 100.0%, *p* = 0.822, Table S3). Alectinib might have better PFS than ceritinib (450 mg once daily) and brigatinib (once daily dosing 90 mg for 1 week and then rose to 180 mg) (*p* = 0.043, Figure 5A). Considering the limited cases in three groups, the conclusion should be interpreted with cautious, and needs further larger scale study to identify. PFS2 of next‐generation TKIs for each patient have been visualized in stacked column graph (Figure 5B).

## 4 DISCUSSION

Fluorescence in situ hybridization (FISH) is the gold standard for gene fusion detection with the disadvantage of poor repeatability requires professional interpretation. IHC is also approved for detecting ALK status due to its good reproducibility and sensitivity. Despite its extensive clinical application, Ventana IHC platform still has some limitations: (1) risk of false negativity; (2) difficulty in distinguishing the fusion partner and concurrent gene mutations. And the above issues can be resolved by NGS.^10^ The previous study demonstrated that IHC and NGS presented a better positive rate (94.5% and 92.7%) than FISH (82.4%), with concordance rate of 87.3% between IHC and NGS. Interestingly, compared to NGS‐negative patients, NGS‐confirmed subjects appeared to have a better PFS, while no difference was observed between FISH‐ and IHC‐positive cases in predicting the efficacy of crizotinib. Notably, TP53 co‐mutation, detected by NGS, have a negative effect on the survival of ALK‐positive patients, further indicating the essential role of NGS application in clinical practice.^11^ Considering this, ALK‐positive patients identified by IHC or NGS were both included in this analysis.

The results of phase III trial, NEJ009, showed that EGFR‐TKI combined with platinum‐based chemotherapy significantly improved PFS and OS in EGFR‐positive NSCLC patients compared with targeted therapy.^12^ Consistent with it, another phase III study in India also came to similar conclusions with enhanced toxicity in NSCLC patients.^13^ After chemotherapy combined with TKI achieved positive results in EGFR‐mutant NSCLC, a question raised: Could chemotherapy synergize TKI in ALK‐positive patients? So far, few relevant prospective studies were conducted. A preclinical study unraveled that the combination of cisplatin and high‐dose crizotinib induced immunogenic cell death in NSCLC cells and effectively controlled the growth of orthotopic models by increasing T lymphocyte infiltration. Furthermore, this combination was able to increase the expression of PD‐1 and PD‐L1 in tumors, and strongly sensitize NSCLC cells to anti‐PD‐1 treatment.^14^ Based on these premises, we analyzed the impact of chemotherapy prior to or in combination with TKI on advanced ALK‐positive NSCLC. And the results from both univariate (Log‐rank test) and multivariate analysis (Cox regression) showed that no improvements were observed. It suggests that chemotherapy might not improve the PFS and reduce the resistance to TKIs. Therefore, considering that the use of combination may lead to more adverse reactions, it should be avoided or adopted cautiously in clinical practice.

Similar to the previous clinical trials and real‐world studies, we also observed that alectinib was superior to crizotinib in prolonging survival of ALK‐positive and TKI‐naive NSCLC, and still had satisfactory effects in patients who progressed to crizotinib treatment. Compared to crizotinib, administration of alectinib also showed better intracranial protection. After the failure of crizotinib treatment, which one works better among next‐generation TKIs? There were no randomized controlled studies for head‐to‐head comparison of next‐generation TKIs in ALK‐positive patients. A retrospective study from China demonstrated that alectinib treatment showed better PFS and higher intracranial efficacy in patients failed to crizotinib treatment. Consistent with it, we also observed that alectinib was better for prolonging PFS than ceritinib and brigatinib in ALK‐positive patients failed to crizotinib treatment. This may be partly attributed to the less BBB penetration of ceritinib than alectinib.^15^ In addition, dose reduction or interruption caused by the higher gastrointestinal toxicity of ceritinib has compromised the intracranial protectivity.^6^, ^16^ Our results provide evidence for comparing the efficacy of ceritinib, brigatinib, and alectinib. A clinical trial (ALTA‐3) comparing alectinib and brigatinib among patients pretreated with crizotinib is ongoing, we look forward to the results.^17^

Do ALK fusion types and EML4‐ALK variants really matter? The landmark study by Heuckman et al proved that differential types of ALK fusion caused differential protein stability and resulted in differential responses to ALK TKIs.^18^ The study by Zhang et al suggested that non‐reciprocal/reciprocal ALK translocation (also known as complex fusion involving EML4‐ALK) predicted the poor efficacy of crizotinib in ALK‐positive patients.^10^ Interestingly, our results showed that EML4 group had a longer PFS than non‐EML4 group after alectinib treatment, but no differences were found between the two groups receiving crizotinib administration. Similarly, a study by Kang et al also suggested that complex fusion involving EML4‐ALK was associated with better survival outcomes.^19^ In addition, they proved some rare fusion, such as GALNT14‐ALK and SETD2‐ALK, may only appear briefly as an intermediate of EML4‐ALK.^19^ Multiple retrospective studies and clinical trials provided further evidence that different EML4‐ALK variants had differential responses and survival outcomes, especially poor outcomes for the “shorter” variants, such as v3a/b and v5a/b.^3^ Preclinical studies by Horn and colleagues clearly demonstrated that the IC50 of all five globally approved next‐generation TKIs was lower than crizotinib in EML4‐ALK V1 and V3 of Ba/F3 cells.^20^ In our analysis, the survival of patients with v1 variant was better than others, and the statistical difference approached significance (HR: 0.56, *p* = 0.071). All the above studies indicated the efficacy prediction of ALK inhibitors and more precise treatment strategies for differential ALK fusions and variants in the future.

Compared with specific variants, concurrent genetic mutations, such as TP53, MYC copy, or CCND1 copy, may yield a negative impact on prognosis.^19^ Also, our analysis showed that the survival of patients with TP53 co‐mutation was poorer than that of those without.^3^, ^11^

## 5 CONCLUSIONS

This retrospective analysis showed that alectinib was superior to crizotinib in ALK‐positive, TKI‐naïve NSCLC with better PFS and higher intracranial penetration. After failure of crizotinib, alectinib might have a better prognosis compared to ceritinib and brigatinib. TP53 co‐mutation had an adverse effect on the survival of ALK‐positive patients. It was worthy to note that the chemotherapy combination seemed unable to improve PFS or reduce the resistance to TKI.

## AUTHORS' CONTRIBUTIONS

YW, RZ, and XL contributed to the conception. YW, PH, and DG contributed to data collection. YW and RZ contributed to data analysis, interpretation, and graphics presentation. YW, RZ, and SS contributed to constructive discussions and manuscript writing. All authors read and approved the final version of the manuscript.

## CONFLICT OF INTEREST

The authors have no conflicts of interest to declare.

## FUNDING INFORMATION

This work was supported by the Youth Innovation Fund of the First Affiliated Hospital of Zhengzhou University (LHGJ20190018 and LHGJ20190030). The funding source had no role in the study design, data collection, analysis, results interpretation, manuscript drafting, and decision to submit the paper for publication.

## ETHICS STATEMENT

The authors are accountable for all aspects of the work in ensuring that questions related to the accuracy or integrity of any part of the work are appropriately investigated and resolved. The study was conducted in accordance with the Declaration of Helsinki (as revised in 2013). The study was approved by the ethics committee of The First Affiliated Hospital of Zhengzhou University and individual consent for this retrospective analysis was waived.

## Supporting information


Figure S1

Table S1–S3

## Data Availability

The datasets used and/or analyzed during the current study are available from the corresponding author on reasonable request.
