# Supplementary figures and images for: Alectinib versus crizotinib in ALK‐positive advanced non‐small cell lung cancer and comparison of next‐generation TKIs after crizotinib failure: Real‐world evidence
Source: Cancer Med. 2022 May 26;11(23):4491–500. doi: 10.1002/cam4.4834 (PMC9741982; doi:10.1002/cam4.4834)

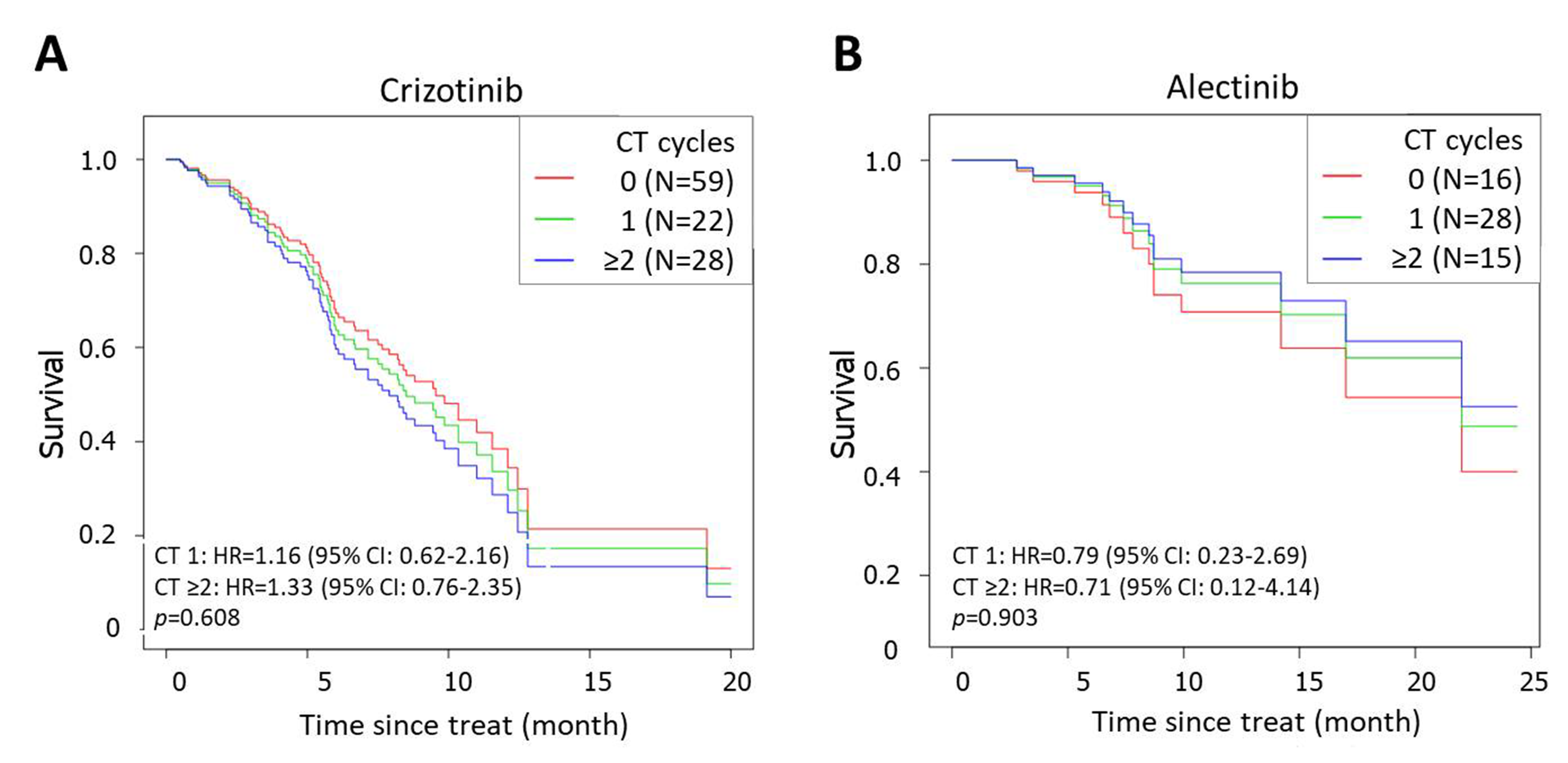

Supplement: Supplementary file 1 — Figure S1 [file CAM4-11-4491-s001.tif]
