# Supplementary material for: Alectinib versus crizotinib in ALK‐positive advanced non‐small cell lung cancer and comparison of next‐generation TKIs after crizotinib failure: Real‐world evidence
Source: Cancer Med. 2022 May 26;11(23):4491–500. doi: 10.1002/cam4.4834 (PMC9741982; doi:10.1002/cam4.4834)
Supplement: Supplementary file 2 — Table S1–S3 [file CAM4-11-4491-s002.docx]

Table S1 Responses to crizotinib and alectinib in ALK-positive NSCLC and the PFS rates

|  | Crizotinib | Alectinib | P value |
| --- | --- | --- | --- |
| ORR, n (%) | 73 (67.0) | 44 (74.6) | 0.306 |
| DCR, n (%) | 104 (95.4) | 58 (98.3) | 0.666 |
| Best objective response, n (%) |  |  |  |
| CR | 0 | 0 |  |
| PR | 73 (67.0) | 44 (74.6) |  |
| SD | 31 (28.4) | 14 (23.7) |  |
| PD | 5 (4.6) | 1 (1.7) |  |
| PFS rate (95% CI) |  |  |  |
| 6 months | 0.85 (0.72-1.00) | 0.93 (0.86-1.00) | 0.015 |
| 12 months | 0.53 (0.28-1.00) | 0.76 (0.56-1.00) | 0.005 |
| 18 months | 0.35 (0.11-1.00) | 0.63 (0.37-1.00) | 0.004 |
| 24 months | 0.15 (0.02-1.00) | 0.44 (0.16-1.00) | 0.004 |

CR, complete response; PR, partial response; SD, stable disease; PD, progressive disease; ORR, objective response rate; DCR, disease control rate

Table S2 Baseline characteristics of patients with ALK-positive NSCLC treated by next-generation TKIs after crizotinib failure.

| Characteristics | Alectinib  （N=19） | Ceritinib  （N=11） | Brigatinib  （N=3） | P-value |
| --- | --- | --- | --- | --- |
| Age, years | 55.0 ± 9.8 | 49.5 ± 12.9 | 41.7 ± 17.6 | 0.141 |
| Sex |  |  |  | 0.929 |
| Male | 11 (57.9%) | 7 (63.6%) | 2 (66.7%) |  |
| Famale | 8 (42.1%) | 4 (36.4%) | 1 (33.3%) |  |
| ECOG PS |  |  |  | 0.775 |
| 0-1 | 15 (78.9%) | 10 (90.9%) | 3 (100.0%) |  |
| 2-3 | 4 (21.1%) | 1 (9.1%) | 0 (0.0%) |  |
| Smoke history |  |  |  | 0.592 |
| No | 15 (78.9%) | 8 (72.7%) | 3 (100.0%) |  |
| Yes | 4 (21.1%) | 3 (27.3%) | 0 (0.0%) |  |
| Histology |  |  |  | 0.068 |
| Adenocarcinoma | 19 (100.0%) | 10 (90.9%) | 2 (66.7%) |  |
| Others | 0 (0.0%) | 1 (9.1%) | 1 (33.3%) |  |
| TNM stage |  |  |  | 0.357 |
| III | 0 (0.0%) | 1 (9.1%) | 0 (0.0%) |  |
| IV | 19 (100.0%) | 10 (90.9%) | 3 (100.0%) |  |
| Detection method |  |  |  | 0.465 |
| IHC | 2 (10.5%) | 1 (9.1%) | 0 (0.0%) |  |
| NGS | 17 (89.5%) | 10 (90.9%) | 3 (100.0%) |  |
| Fusion type |  |  |  | 0.135 |
| EML4-ALK | 16 (84.2%) | 6 (54.5%) | 1 (33.3%) |  |
| Non-EML4-ALK | 1 (5.3%) | 3 (27.3%) | 1 (33.3%) |  |
| Complex fusion | 0 (0.0%) | 1 (9.1%) | 1 (33.3%) |  |

ECOG PS: Eastern Cooperative Oncology Group performance status, CT: Chemotherapy,

Table S3 Responses and outcomes of next-generation TKIs in ALK-positive NSCLC

|  | Alectinib | Ceritinib | Brigatinib | P-value |
| --- | --- | --- | --- | --- |
| ORR, n (%) | 5 (26.3%) | 3 (27.3%) | 0 (0.0%) | 0.589 |
| DCR, n (%) | 18 (94.7%) | 10 (90.9%) | 3 (100.0%) | 0.822 |
| Best responses |  |  |  |  |
| CR | 0 | 0 | 0 |  |
| PR | 5 (26.3%) | 3 (27.3%) | 0 (0.0%) |  |
| SD | 13 (68.4%) | 7 (63.6%) | 3 (100.0%) |  |
| PD | 1 (5.3%) | 1 (9.1%) | 0 (0.0%) |  |

CR, complete response; PR, partial response; SD, stable disease; PD, progressive disease; ORR, objective response rate; DCR, disease control rate
